# A New Cation‐Ordered Structure Type with Multiple Thermal Redistributions in Co_2_InSbO_6_

**DOI:** 10.1002/anie.202203062

**Published:** 2022-04-21

**Authors:** Kunlang Ji, Elena Solana‐Madruga, Midori Amano Patino, Yuichi Shimakawa, J. Paul Attfield

**Affiliations:** ^1^ Centre for Science at Extreme Conditions (CSEC) School of Chemistry University of Edinburgh Mayfield Road Edinburgh EH9 3FD UK; ^2^ Dpto. Q. Inorgánica Universidad Complutense de Madrid Avda. Complutense sn 28040 Madrid Spain; ^3^ Institute for Chemical Research Kyoto University Uji Kyoto 611-0011 Japan

**Keywords:** Corundum Types, High-Pressure Chemistry, Magnetic Properties, Solid-State Structures

## Abstract

Cation ordering in solids is important for controlling physical properties and leads to ilmenite (FeTiO_3_) and LiNbO_3_ type derivatives of the corundum structure, with ferroelectricity resulting from breaking of inversion symmetry in the latter. However, a hypothetical third ABO_3_ derivative with *R*32 symmetry has never been observed. Here we show that Co_2_InSbO_6_ recovered from high pressure has a new, ordered‐*R*32 A_2_BCO_6_ variant of the corundum structure. Co_2_InSbO_6_ is also remarkable for showing two cation redistributions, to (Co_0.5_In_0.5_)_2_CoSbO_6_ and then Co_2_InSbO_6_ variants of the ordered‐LiNbO_3_ A_2_BCO_6_ structure on heating. The cation distributions change magnetic properties as the final ordered‐LiNbO_3_ product has a sharp ferrimagnetic transition unlike the initial ordered‐*R*32 phase. Future syntheses of metastable corundum derivatives at pressure are likely to reveal other cation‐redistribution pathways, and may enable ABO_3_ materials with the *R*32 structure to be discovered.

## Graphical abstract

Corundum‐derived Co_2_InSbO_6_ undergoes an unprecedented sequence of cation rearrangements on heating, from a previously unreported ordered‐*R*32 type distribution to two successive ordered‐LiNbO_3_ type arrangements.

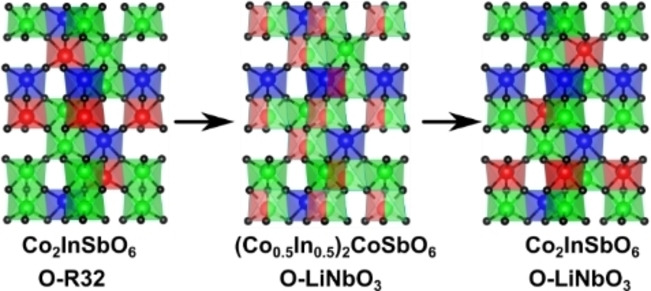

Cation ordering within extended oxide structures is an important way to control physical properties such as introduction of ferroelectricity and multiferroism from arrangements that break inversion symmetry.[^1^, ^2^] This is notably illustrated by the corundum (α‐Al_2_O_3_) type A_2_O_3_ structure which has a simple centrosymmetric arrangement with rhombohedral space group symmetry. A_2_O_9_ dimer units of two octahedra sharing a common face are separated by single vacant octahedra to form AA_AA chains (Figure 1). Two cation‐ordered ABO_3_ derivatives are known—the ilmenite (FeTiO_3_) and LiNbO_3_ types with centric and acentric *R*3*c* symmetry respectively. Both have AB cation pairs in the dimer units with antiparallel AB_BA alignment in ilmenite but parallel AB_AB order leading to polarity in the LiNbO_3_ type. It is intriguing to note that a third ABO_3_ cation ordering type is also possible within the corundum unit cell as shown in Figure 1. This structure has *R*32 symmetry with AA_BB chains of dimer pairs, and no examples have been reported. Further cation ordering within the ABO_3_ structures leads to A_2_BCO_6_ derivatives, referred to in the literature as “ordered‐ABO_3_” types. These were first found in Li_2_MTeO_6_ phases which adopt the ordered‐LiNbO_3_ structure for M=Zr and Hf,^[3]^ and the ordered‐ilmenite type for M=Ge.^[4]^ The “ordered‐*R*32” A_2_BCO_6_ derivative of the *R*32‐type has not been reported. These three A_2_BCO_6_ arrangements all have *R*3 symmetry with four symmetry‐independent octahedral cation sites. This structure is known as the Ni_3_TeO_6_ (NTO) type and represents a special case of all three A_2_BCO_6_ types (Figure 1) where A=B=Ni and C=Te.

**Figure 1:**
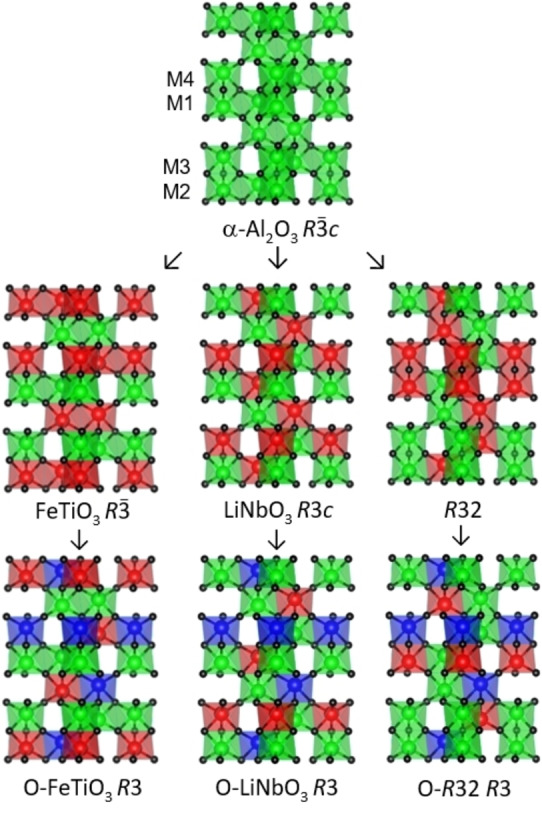
Crystal structures of the corundum type A_2_O_3_ structure (top), and ABO_3_ (middle row) and A_2_BCO_6_ (“ordered‐ABO_3_”, bottom row) derivatives obtained through cation ordering. Symmetry descents are indicated by the arrows and space groups are shown. Colours indicate the cation occupancies of octahedra in each structure (A/B/C=green/red/blue). The four site labels shown by the A_2_O_3_ structure are used throughout this paper, where M4=Sb in Co_2_InSbO_6_.

The non‐centrosymmetric cation ordered corundum derivatives offer a rich variety of properties[^5^, ^6^, ^7^] according to their point group symmetry.^[8]^ *R*3*c* LiNbO_3_‐types (*C*_3v_ point group) have allowed polar and piezoelectric activity, and LiNbO_3_ itself is an important ferroelectric, piezoelectric and non‐linear optical material.^[9]^ The *R*32 ABO_3_ structure (*D*_3_) is notable as belonging to the class of space groups that are non‐polar but allow enantiomorphic and piezoelectric activity, so discovered examples would be of great interest. The ordered A_2_BCO_6_ (NTO‐type) structures (*C*_3_) have allowed polar, chiral and piezoelectric properties arising from their *R*3 symmetry. Further coupling of these structural orders to magnetism (multiferroism) can be introduced by use of magnetic cations that adopt long‐range spin orders at low temperatures.^[10]^ High pressure is often used to stabilise these acentric cation‐ordered corundum derivatives, for example, MnTiO_3_ changes from a centric ilmenite type at ambient pressure to an acentric LiNbO_3_‐type high‐pressure polymorph where weak ferromagnetism offers a mechanism for multiferroic switching.^[11]^ Mn(Fe_0.5_M_0.5_)O_3_ (M=Nb, Ta) are further examples of ABO_3_ LiNbO_3_‐types, with Fe/M disorder.^[12]^ Within the *R*3 A_2_BCO_6_ types, β‐Mn_2_InSbO_6_ has the ordered‐ilmenite arrangement^[13]^ while M_2_ScSbO_6_ (M=Mn,^[14]^ Co,^[15]^ Ni^[16]^) and Mn_2_FeWO_6_ ^[17]^ are ordered‐LiNbO_3_ types. Mn_2_FeMoO_6_ recovered from high pressure synthesis has an ordered‐LiNbO_3_ structure but this changes to an ordered‐ilmenite type on heating and the stabilisation of these two types was rationalised from band structure calculations.^[18]^ Magnetoelectric effects are reported in Ni_3_TeO_6_[^19^, ^20^, ^21^] and ternary NTO‐type analogues have recently been discovered for A_3_TeO_6_ (A=Mn, Co)^[22]^ and Mn_3_WO_6_ ^[23]^ at high pressure. In this communication, we report the synthesis of the new double‐corundum material Co_2_InSbO_6_ and thermal cation redistributions of unprecedented complexity from the previously unobserved ordered‐*R*32 type to two different ordered‐LiNbO_3_ types.

A mixture of CoO, In_2_O_3_ and Sb_2_O_5_ in stoichiometric proportions for product Co_2_InSbO_6_ was treated under high pressure and temperature conditions using a multi‐anvil apparatus. Further details are in Supporting Information. A sample recovered from 6 GPa and 1373 K was found to contain a CaCl_2_‐type product with an orthorhombic structure that is unrelated to the corundum types and characterisation of this phase is described in Supporting Information. Synthesis under 8 GPa and 1373 K led to a recovered Co_2_InSbO_6_ product with *R*3 symmetry (lattice parameters *a*=5.2882(3) Å and *c*=14.029(1) Å) consistent with A_2_BCO_6_ structures shown in Figure 1.

Synchrotron powder X‐ray diffraction data from the Co_2_InSbO_6_ sample were collected in situ while heating from 300 to 1073 K to determine the structure and any thermal changes. Refinement of the recovered Co_2_InSbO_6_ product structure at 300 K (fit and results in Supporting Information) gave cation site occupancies M1=Co_0.3_In_0.7_, M2=Co_0.7_In_0.3_, M3=Co and M4=Sb. Remarkably, the two Co‐rich sites M2 and M3 are present in the same dimer units so that the cation distribution is close to the ordered‐*R*32 type rather than the ordered‐ ilmenite or LiNbO_3_ types. This is an important structural discovery given that no ordered‐*R*32 A_2_BCO_6_ or *R*32 ABO_3_ structures have been reported amongst many known corundum‐derived phases. The present compound thus represents a new structural type within the corundum group (Figure 1).

Comparison of the variable temperature patterns in Figure 2 shows that Co_2_InSbO_6_ persists as a *R*3 corundum‐derived material up to 1073 K but changes in peak positions and intensities near 900 K reveal structural rearrangement. Initial unconstrained fits (summarised in Supporting Information) demonstrated that while one cation site (M4) remains occupied by Sb throughout, Co/In occupancies at the other three sites change with temperature. Thermal variations of the cell parameters, M1–M3 site occupancies and M−O bond lengths from final refinements constrained to the overall Co_2_InSbO_6_ stoichiometry are shown in Figure 3. Further results are tabulated in Supporting Information.

**Figure 2:**
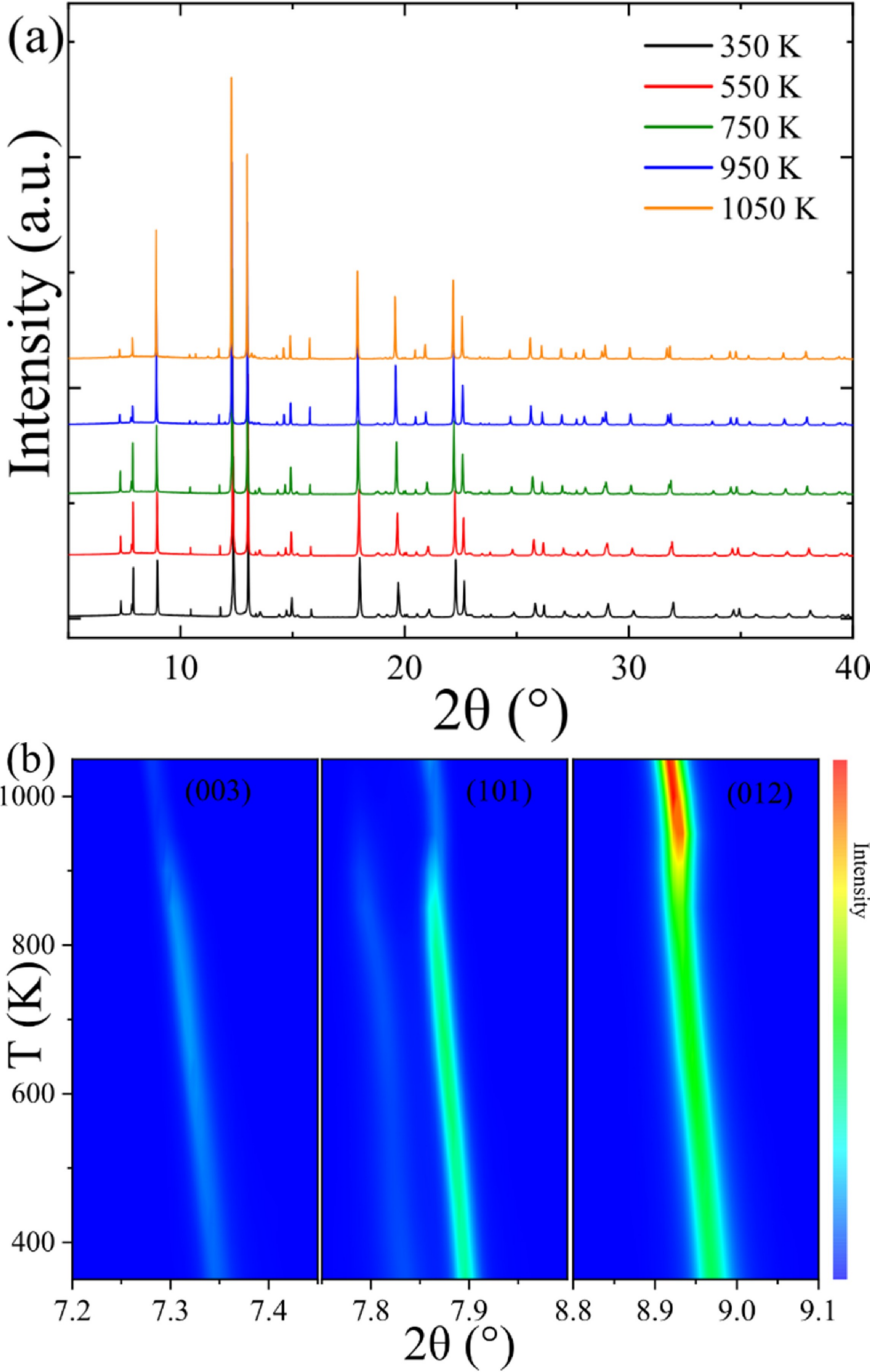
a) Selected powder X‐ray diffraction data from the high pressure Co_2_InSbO_6_ sample collected in situ while heating from 300 to 1073 K. b) Diffraction intensity map for low‐angle (003), (101) and (012) peaks. Changes between 850 and 950 K reflect the evolving cation distributions.

**Figure 3:**
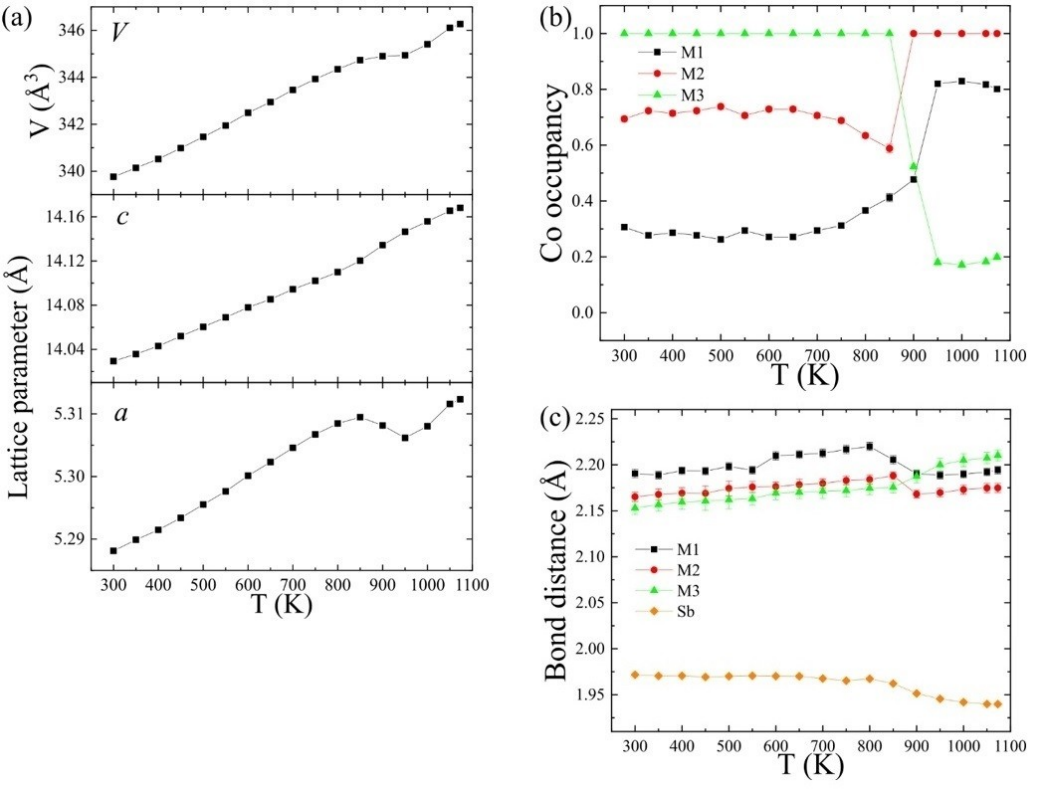
Refined X‐ray structure parameters from Co_2_InSbO_6_ while heating from 300 to 1073 K. a) Lattice parameters and cell volume showing the structural anomaly between 850 and 950 K. b) Co occupancies at M1, M2 and M3 sites revealing Co/In intersite rearrangements. c) Average M−O bond lengths for each MO_6_ octahedron where M1–M3 are occupied by Co/In and Sb is at the M4 site.

Refined cell parameters for Co_2_InSbO_6_ in Figure 3a show a change in curvature on heating above 650 K and a dramatic lattice anomaly between 850 and 950 K. A negative expansion in the *a* parameter, a small excess positive expansion in *c*, and almost zero volume expansion over this interval are observed. Corresponding changes in the Co/In occupancies at the M1–M3 sites in Figure 3b reveal that two successive cation rearrangements occur on heating. Cation populations remain constant from 300 up to 650 K, reflecting the kinetic sluggishness of migration on the timescale of the X‐ray diffraction experiment. Above 650 K the M3 site remains fully occupied by Co, but the Co/In occupancies of the M1 and M2 sites gradually converge and are estimated to become equal at *T*_c1_=880 K from a mean field fit to the occupancy difference (shown in Supporting Information). This transition corresponds to a change between different A_2_BCO_6_ types in Co_2_InSbO_6_: from the ordered *R*32‐type in the recovered sample to an ordered‐LiNbO_3_ arrangement upon heating. In the latter structure where the two A sites (M1 and M2) have identical disordered Co_0.5_In_0.5_ compositions at *T*_c1_. This transition highlights the instability of the *R*32 cation distribution and ordered‐*R*32 derivative at ambient conditions. Furthermore, the ordered‐LiNbO_3_ phase (Co_0.5_In_0.5_)_2_CoSbO_6_ shows a thermal instability immediately above *T*_c1_ as In from the M1 and M2 sites rapidly exchanges with Co from the M3 site between *T*_c1_ and *T*_c2_≈950 K. Above *T*_c2_, the cation distribution is close to another A_2_BCO_6_ ordered‐LiNbO_3_ type, with A sites having M1≈80 % and M2=100 % Co. Hence the discovered sequence of structural changes (showing ideal A_2_BCO_6_ cation site occupancies as displayed in the Table of Contents graphic) is;

(1) $$\;\mathrm{Co}{}_{2}\mathrm{InSbO}{}_{6}\overset{T{}_{c1}=880\;K}{\underset{}{\to }}\;\left(\mathrm{Co}{}_{0.5}\mathrm{In}{}_{0.5}\right){}_{2}\mathrm{CoSbO}{}_{6}\overset{T{}_{c2}=950\;K}{\underset{}{\to }}\;\mathrm{Co}{}_{2}\mathrm{InSbO}{}_{6}O\text{-}R32\;O\text{-}\mathrm{LiNbO}{}_{3}\;O\text{-}\mathrm{LiNbO}{}_{3}$$

These observations demonstrate that the overall transformation of Co_2_InSbO_6_ from an ordered‐*R*32 to an ordered‐LiNbO_3_ polymorph occurs via a cation‐disordered (Co_0.5_In_0.5_)_2_CoSbO_6_ ordered‐LiNbO_3_ intermediate phase. The second transition, from a cation‐disordered to a cation‐ordered structure on heating, is unusual given the loss of configurational entropy and evidences likely metastability of the intermediate (Co_0.5_In_0.5_)_2_CoSbO_6_ phase.

Average metal–oxygen bond lengths (Figure 3c) are consistent with the Co/In occupancy rearrangements, given the cation sizes (6‐coordinate ionic radii are Co^2+^=0.745, In^3+^=0.80, and Sb^5+^=0.60 Å).^[24]^ M1−O and M2−O distances both decrease on heating from 850 to 950 K as their Co‐populations increase, while an increase in the M3−O distance reflects the almost complete replacement of Co^2+^ by In^3+^. The similar sizes and charges of Co^2+^ and In^3+^ cations allow the changing cation distributions at M1‐M3 sites while the smaller and more highly charged Sb^5+^ occupies only the M4 site throughout.

Interplay between cation sizes and charges provides a likely explanation for the observed sequence of structures for Co_2_InSbO_6_. Efficient cation packing is favoured under the high pressure (8 GPa) at which the initial sample was synthesised. The ordered‐*R*32 structure with Co^2+^Co^2+^_In^3+^Sb^5+^ chains of cation pairs is thus stabilised as Co^2+^ (0.745 Å) is similar in size to the average (0.70 Å) of the larger In^3+^ and smaller Sb^5+^ cations. Thermal relaxation at ambient pressure leads to (Co_0.5_In_0.5_)^2.5+^Co^2+^_(Co_0.5_In_0.5_)^2.5+^Sb^5+^ and then to Co^2+^In^3+^_Co^2+^Sb^5+^ sequences of cation pairs in the successive ordered‐LiNbO_3_ type products. This reduces electrostatic repulsions between cations in the dimer pairs which becomes more significant at ambient pressure where packing constraints are less important. Repulsions between cation charges *q_i_* in the dimer pairs can be quantified in a simple nearest‐neighbour approximation as *E*=*q*_A_ *q*_B_+*q*_C_ *q*_D_ for AB_CD cation order in the chains assuming fixed cation‐cation separations. The sequence of structures shown above as (1) have *E*=19→17.5→16 and the Co^2+^In^3+^_Co^2+^Sb^5+^sequence in the final ordered‐LiNbO_3_ product has the lowest possible electrostatic repulsion energy within the family of A_2_BCO_6_ structures (Figure 1), as the ordered‐ilmenite alternative would have greater repulsion across Co^2+^In^3+^_Sb^5+^Co^2+^ pairs. This lowering of cation‐cation repulsion is consistent with the decrease in thermal expansion of *a* and *V* cell parameters on heating across the two transitions seen in Figure 3a.

The effects of the cation rearrangement on the magnetic properties of Co_2_InSbO_6_ were explored by comparing the original sample recovered from high pressure with ordered‐*R*32 structure type (OR32 sample), with a sample subsequently heated to 1073 K having the final ordered‐LiNbO_3_ cation arrangement (OLN sample). Magnetic susceptibility measurements show that both samples are Curie–Weiss paramagnets at high temperatures (Figure 4), with effective paramagnetic moment *μ*_eff_=5.19 μ_B_ per Co^2+^ and Weiss temperature *θ*=−43 K for the OR32 sample, and *μ*_eff_=5.30 μ_B_ and *θ*=−106 K for OLN. The moments are in excess of spin‐only values showing that strong orbital contributions are present, and similar values up to ≈5.20 μ_B_ have been reported for other Co^2+^ oxides such as Co_2_ScSbO_6_.^[15]^ Negative values of *θ* indicate that dominant spin‐spin interactions are antiferromagnetic. Both samples show deviation of the susceptibility above the Curie–Weiss variation at temperatures below ≈65 K suggesting antiparallel but ferrimagnetic spin alignments, given the negative values of *θ*. The OR32 sample shows no discontinuity or divergence of zero‐field cooled and field cooled (ZFC and FC) susceptibilities, which suggest short range ferrimagnetism. However, the OLN sample has a sharp Curie transition at *T*_C_=65 K, similar to *T*_C_=59 K for isostructural ferrimagnetic Co_2_ScSbO_6_.^[15]^ This contrasting behaviour reflects a key difference in cobalt spin distributions in the two structures. In the *R*32 structure, the Co spins are located in dimers which results in frustration between successive dimer layers, but in the ordered‐LiNbO_3_ structure the spins are distributed in a less frustrated, three‐dimensional network. Both samples have susceptibility peaks at 15 K indicative of a possible antiferromagnetic or a spin‐glass transition. The latter could result from the 20–25 % Co/In disorder between two sites observed for both samples. Neutron diffraction will be needed to confirm the spin orders or their absence. Magnetization‐field loops show substantial magnetization for the two samples at low temperatures (Figure 4). The moments at 5 K and 5 T approach 2 and 1 μ_B_ per Co_2_InSbO_6_ formula unit for OR32 and OLN samples, respectively. The OR32 sample exhibits a small hysteresis at 5 K (remnant magnetization *M*_r_=0.04 μ_B_ and coercive field *B*_c_=0.03 T) but hysteresis for the OLN sample is more substantial (*M*_r_=0.12 μ_B_ and *B*_c_=0.16 T), consistent with the well‐defined ferrimagnetic transition for this phase.

**Figure 4:**
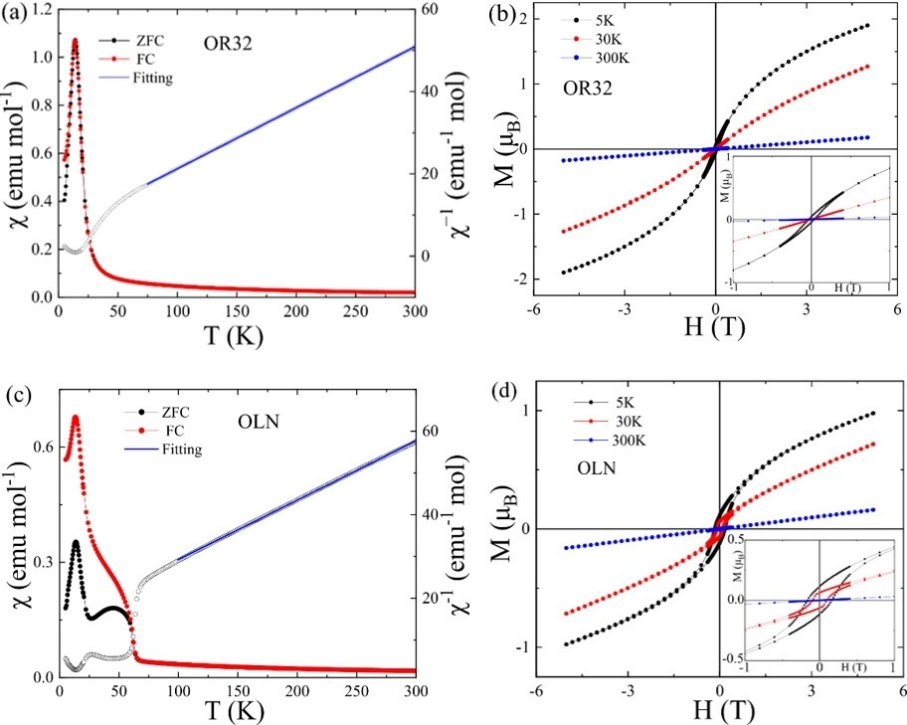
Magnetic measurements for a) and b) the OR32, and c) and d) the OLN, samples of Co_2_InSbO_6_. a) and c) ZFC and FC susceptibilities and inverse ZFC susceptibilities with high‐temperature Curie–Weiss fits. b) and d) Magnetisation‐field loops with insets showing low field regions.

These results demonstrate that a new A_2_BCO_6_ ordered‐*R*32 variant of the corundum structure is stabilised in Co_2_InSbO_6_ prepared at high pressure. This discovery of dimer units containing the same cations is unprecented in ABO_3_ or A_2_BCO_6_ corundum derivatives and likely results from similar average cation sizes in the Co_2_O_9_ and InSbO_9_ dimer units minimising volume at pressure. High pressure may thus enable discovery of ABO_3_ phases with the as‐yet unreported *R*32 structure. These would be of interest for the stabilization of spin‐dimer materials with A/B=magnetic/non‐magnetic cation combinations. Co_2_InSbO_6_ is also remarkable for showing an unprecedented sequence of two cation rearrangements on heating: first to (Co_0.5_In_0.5_)_2_CoSbO_6_ and then to Co_2_InSbO_6_ phases with the ordered‐LiNbO_3_ structure. These rearrangements reduce cation‐cation repulsions and favour the ordered‐LiNbO_3_ structure that is often observed in corundum‐derived A_2_BCO_6_ materials. The cation distributions change magnetic properties as the final ordered‐LiNbO_3_ product has a sharp ferrimagnetic transition at 65 K whereas the initial ordered‐*R*32 phase has a broader feature indicative of short‐range spin ordering. Both samples have susceptibility peaks at 15 K indicative of an antiferromagnetic or spin‐glass transition. All of the Co_2_InSbO_6_ phases have acentric *R*3 symmetry, and so are of interest for future exploration of acentric and multiferroic properties.

In conclusion, Co_2_InSbO_6_ and previously‐reported Mn_2_FeMoO_6_ ^[18]^ demonstrate that high pressure may be used to recover metastable cation arrangements within the corundum family that can be thermally relaxed to new structures. This enables dependence of physical properties on the cation‐ordering patterns to be explored. Further syntheses of high‐pressure corundum derivatives are likely to reveal other cation‐redistribution pathways, and may enable ABO_3_ materials with the as‐yet unreported *R*32 structure type to be discovered.

## Conflict of interest

The authors declare no conflict of interest.

## Supporting information

As a service to our authors and readers, this journal provides supporting information supplied by the authors. Such materials are peer reviewed and may be re‐organized for online delivery, but are not copy‐edited or typeset. Technical support issues arising from supporting information (other than missing files) should be addressed to the authors.

Supporting Information

## Data Availability

The data that support the findings of this study are openly available in Edinburgh DataShare at https://datashare.ed.ac.uk/handle/10283/838.

## References

[1] J. M. Rondinelli , C. J. Fennie , Adv. Mater. 2012, 24, 1961–1968. 22488734 10.1002/adma.201104674

[2] R. Shankar P N , F. Orlandi , P. Manuel , W. Zhang , P. S. Halasyamani , A. Sundaresan , Chem. Mater. 2020, 32, 5641–5649.

[3] J. Choisnet , A. Rulmont , P. Tarte , J. Solid State Chem. 1988, 75, 124–135.

[4] P. M. Woodward , A. W. Sleight , L. S. Du , C. P. Grey , J. Solid State Chem. 1999, 147, 99–116.

[5] R. Shankar P N , S. Mishra , S. Athinarayanan , APL Mater. 2020, 8, 040906.

[6] M. Ye , D. Vanderbilt , Phys. Rev. B 2016, 93, 134303.

[7] H. Niu , M. J. Pitcher , A. J. Corkett , S. Ling , P. Mandal , M. Zanella , K. Dawson , P. Stamenov , D. Batuk , A. M. Abakumov , C. L. Bull , R. I. Smith , C. A. Murray , S. J. Day , B. Slater , F. Cora , J. B. Claridge , M. J. Rosseinsky , J. Am. Chem. Soc. 2017, 139, 1520–1531. 28013545 10.1021/jacs.6b11128

[8] P. S. Halasyamani , K. R. Poeppelmeier , Chem. Mater. 1998, 10, 2753–2769.

[9] K. K. Wong, *Properties of Lithium Niobate*, INSPEC, UK, **2002**.

[10] G. H. Cai , M. Greenblatt , M. R. Li , Chem. Mater. 2017, 29, 5447–5457.

[11] A. M. Arévalo-López , J. P. Attfield , Phys. Rev. B 2013, 88, 104416.

[12] M.-R. Li , D. Walker , M. Retuerto , T. Sarkar , J. Hadermann , P. W. Stephens , M. Croft , A. Ignatov , C. P. Grams , J. Hemberger , I. Nowik , P. S. Halasyamani , T. T. Tran , S. Mukherjee , T. S. Dasgupta , M. Greenblatt , Angew. Chem. Int. Ed. 2013, 52, 8406–8410; 10.1002/anie.201302775 23813619

[13] A. M. Arévalo-López , E. Solana-Madruga , E. P. Arévalo-López , D. Khalyavin , M. Kepa , A. J. Dos Santos-García , R. Sáez-Puche , J. P. Attfield , Phys. Rev. B Phys. Rev. B 2018, 98, 214403.

[14] E. Solana-Madruga , A. Dos Santos-García , A. Arévalo-López , D. Ávila-Brande , C. Ritter , J. P. Attfield , R. Sáez-Puche , Dalton Trans. 2015, 44, 20441–20448. 26511286 10.1039/c5dt03445k

[15] K. Ji , E. Solana-Madruga , A. M. Arévalo-López , P. Manuel , C. Ritter , A. Senyshyn , J. P. Attfield , Chem. Commun. 2018, 54, 12523–12526. 10.1039/c8cc07556e 30345452

[16] S. A. Ivanov , R. Mathieu , P. Nordblad , R. Tellgren , C. Ritter , E. Politova , G. Kaleva , A. Mosunov , S. Stefanovich , M. Weil , Chem. Mater. 2013, 25, 935–945.

[17] M.-R. Li , M. Croft , P. W. Stephens , M. Ye , D. Vanderbilt , M. Retuerto , Z. Deng , C. P. Grams , J. Hemberger , J. Hadermann , W.-M. Li , C.-Q. Jin , F. O. Saouma , J. I. Jang , H. Akamatsu , V. Gopalan , D. Walker , M. Greenblatt , Adv. Mater. 2015, 27, 2177–2181. 25677612 10.1002/adma.201405244

[18] M.-R. Li , M. Retuerto , P. W. Stephens , M. Croft , D. Sheptyakov , V. Pomjakushin , Z. Deng , H. Akamatsu , V. Gopalan , J. Sánchez-Benítez , F. O. Saouma , J. I. Jang , D. Walker , M. Greenblatt , Angew. Chem. Int. Ed. 2016, 55, 9862–9867; 10.1002/anie.201511360 27203790

[19] Y. S. Oh , S. Artyukhin , J. J. Yang , V. Zapf , J. W. Kim , D. Vanderbilt , S. W. Cheong , Nat. Commun. 2014, 5, 3201. 24469350 10.1038/ncomms4201

[20] J. W. Kim , S. Artyukhin , E. D. Mun , M. Jaime , N. Harrison , A. Hansen , J. J. Yang , Y. S. Oh , D. Vanderbilt , V. S. Zapf , S.-W. Cheong , Phys. Rev. Lett. 2015, 115, 137201. 26451580 10.1103/PhysRevLett.115.137201

[21] M. O. Yokosuk , A. al-Wahish , S. Artyukhin , K. R. O′Neal , D. Mazumdar , P. Chen , J. J. Yang , Y. S. Oh , S. A. McGill , K. Haule , S.-W. Cheong , D. Vanderbilt , J. L. Musfeldt , Phys. Rev. Lett. 2016, 117, 147402. 27740819 10.1103/PhysRevLett.117.147402

[22] E. Solana-Madruga , C. Aguilar-Maldonado , C. Ritter , M. Huvé , O. Mentré , J. P. Attfield , A. M. Arévalo-López , Chem. Commun. 2021, 57, 2521–2514. 10.1039/d0cc07487j 33538279

[23] M. R. Li , E. E. McCabe , P. W. Stephens , M. Croft , L. Collins , S. V. Kalinin , Z. Deng , M. Retuerto , A. Sen Gupta , H. Padmanabhan , V. Gopalan , C. P. Grams , J. Hemberger , F. Orlandi , P. Manuel , W.-M. Li , C.-Q. Jin , D. Walker , M. Greenblatt , Nat. Commun. 2017, 8, 2037. 29229914 10.1038/s41467-017-02003-3 PMC5725588

[24] R. D. Shannon , Acta Crystallogr. Sect. A 1976, 32, 751–767.

