# Supplementary material for: A New Cation‐Ordered Structure Type with Multiple Thermal Redistributions in Co2InSbO6
Source: Angew Chem Int Ed Engl. 2022 Apr 21;61(27):e202203062. doi: 10.1002/anie.202203062 (PMC9321074; doi:10.1002/anie.202203062)
Supplement: Supplementary file 1 — Supporting Information [file ANIE-61-0-s001.pdf]

Angewandte Chemie
Eine Zeitschrift der Gesellschaft Deutscher Chemiker

Supporting Information

A New Cation-Ordered Structure Type with Multiple Thermal Redistributions in Co_2InSbO_6

K. Ji, E. Solana-Madruga, M. A. Patino, Y. Shimakawa, J. P. Attfield**

Supplementary Information

Experimental Methods

HPHT Synthesis

Samples were prepared by high-pressure high temperature (HPHT) synthesis using a multi-anvil apparatus. The precursor mixtures were prepared by grinding together stoichiometric proportions of binary oxides CoO, In_2O_3 and Sb_2O_5 under acetone followed by packed into a Pt capsule and assembled into a cubic-anvil-type high-pressure apparatus. Samples were compressed and then heated at conditions shown below, and then cooled to room temperature before pressure was slowly released.

$CaCl_2$-type phase A sample recovered from 6 GPa and 1373 K was found to contain a $CaCl_2$-type product with an orthorhombic *Pnnm* structure that has only one symmetry-unique cation site and is unrelated to the corundum types. A stoichiometric '$(Co_{0.5}In_{0.25}Sb_{0.25})O_2$' product would require Co^{4+} which is unlikely given the lack of a strong oxidising agent in the high pressure reaction, but a $((Co_{1-x}In_x)_{0.5}Sb_{0.5})O_2$ composition based on Co^{3+} is more plausible. Refinement of the Co/In ratio in the fit to powder X-ray data (Fig. S1) gave a Co-poor composition $(Co_{0.17}In_{0.33}Sb_{0.5})O_2$ consistent with observation of Co-based secondary phases (CoO and $CoSb_2O_6$). Crystal structure information and magnetic results for this phase are respectively in Table S1 and Fig. S2.

Corundum-related Co_2InSbO_6 phase Synthesis conditions for corundum-related Co_2InSbO_6 were 8 GPa with heating at 1373 K for one hour followed by slow cooling to room temperature.

Powder X-ray diffraction

The obtained HPHT reaction products were initially characterised by powder X-ray diffraction collected with a Bruker Advanced D8 diffractometer. Detailed crystal information of Co_2InSbO_6 was analysed using synchrotron X-ray diffraction (SXRD) data collected at BL02B2 in Spring-8 (λ = 0.5996871 Å). A 0.1 mm quartz glass capillary was used as sample holder to minimize absorption and was rotated during the measurements. SXRD data were collected between 300 and 1073 K with an interval of 50 K. The crystal structure parameters were refined by the Rietveld method using the Fullprof suite.[1] Inspection of the data showed that no *c*-glide plane is present and comparison of fits to 300 K SXRD data in centric $R\overline{3}$ (residuals; Rp = 7.7%, Rwp = 11.5%, RBragg = 6.1%, RF = 3.6%) and acentric $R3$ (Rp = 6.4%, Rwp = 10.1%, RBragg = 4.3%, RF = 2.1%) demonstrated that the latter gives a significantly better fit, and this space group was used throughout subsequent analysis. Results of initial unconstrained cation site refinements to data at all temperatures are shown in Fig. S3 and the deductions that were drawn and used as constraints in the final refinements are described in the caption. Results from final refinements where cation occupancies were constrained to the ideal Co_2InSbO_6 composition are shown in Figs S4-S6 and Tables S2-S5.

Magnetic property measurement

Magnetic property measurements of $(Co_{0.17}In_{0.33}Sb_{0.5})O_2$ were carried out using a Quantum Design PPMS while Co_2InSbO_6 samples were measured using a Quantum Design MPMS-XL SQUID magnetometer. The magnetic susceptibility data were collected between under an applied field of 1000 Oe.

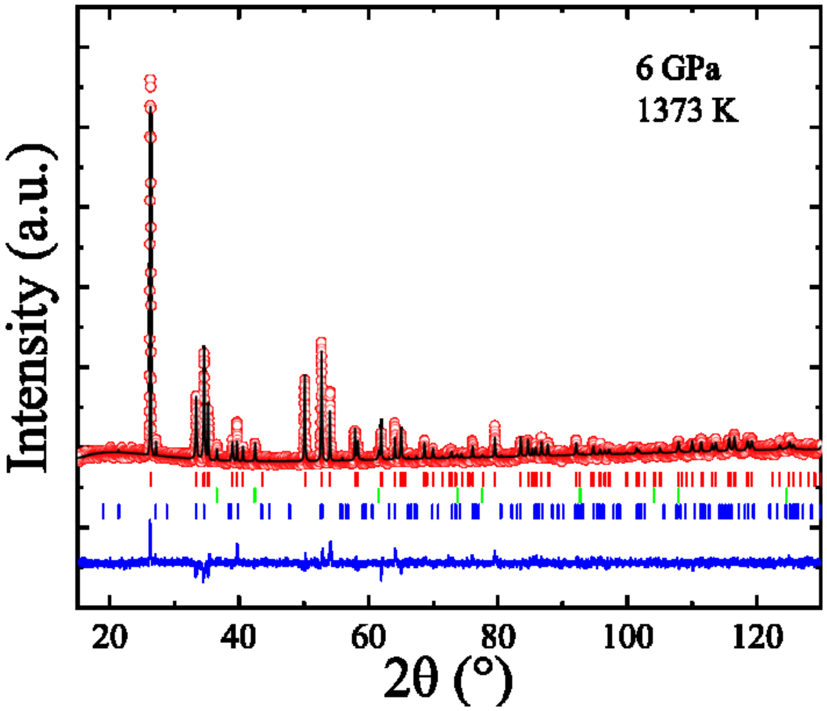

Fig. S1 Rietveld fit to lab X-ray diffraction data of $(Co_{0.17}In_{0.33}Sb_{0.5})O_2$ showing a $CaCl_2$-type tetragonal rutile structure. Impurities CoO (6.8 wt %) and $CoSb_2O_6$ (2.2 wt %) are shown by green and blue tick marks.

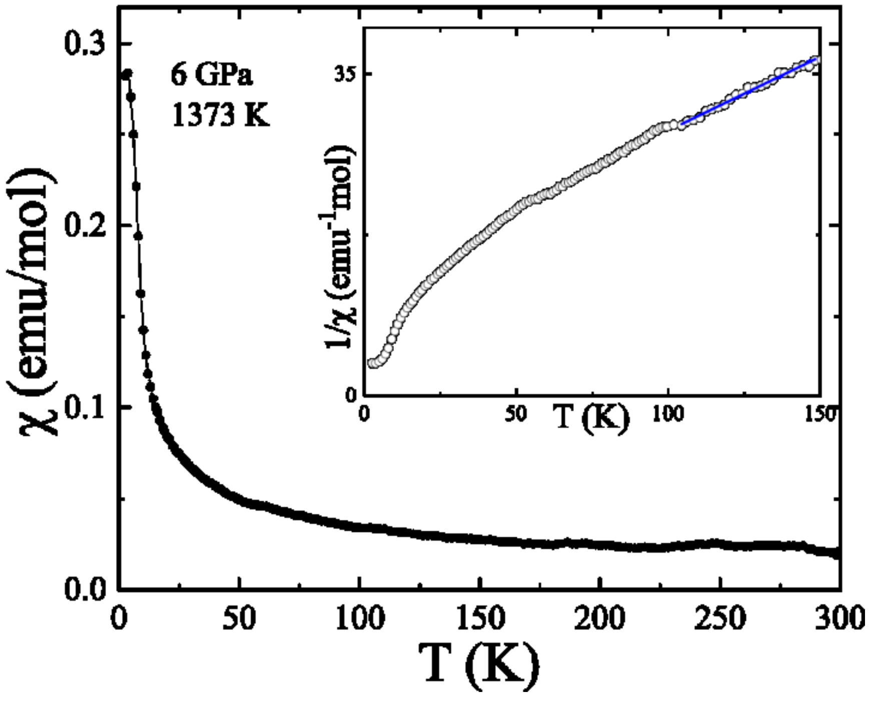

Fig. S2 ZFC susceptibility of $(Co_{0.17}In_{0.33}Sb_{0.5})O_2$ collected from 300 K down to 2 K, showing paramagnetic behavior with a possible spin freezing transition near base temperature. Inset shows inverse susceptibility and a Curie-Weiss fit between 100 K and 150 K which gives the effective paramagnetic moment of μ_{eff} = 5.00 μ_B per Co. This shows that Co^{2+}/ Co^{3+} ions present are in high spin states with orbital contributions to the moment.

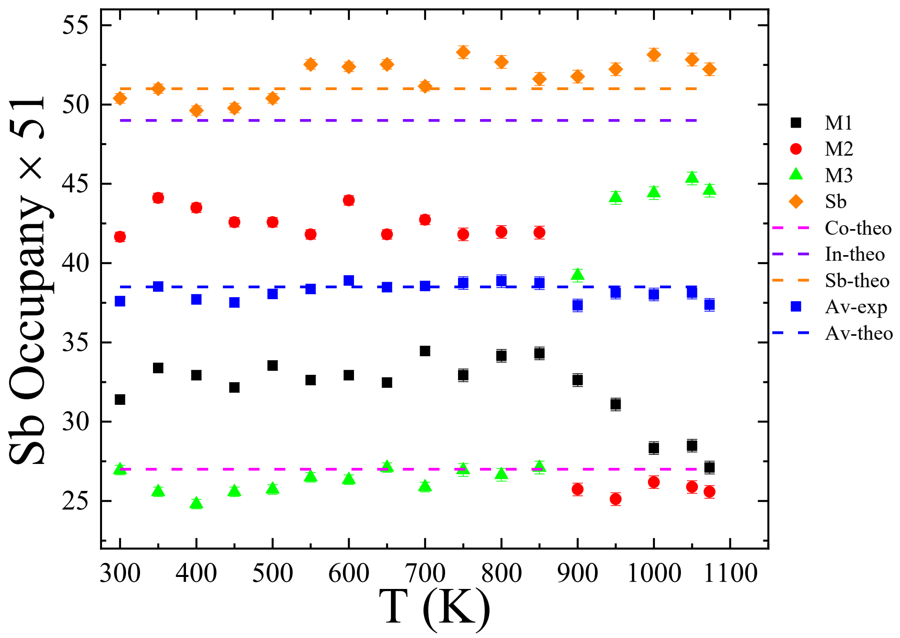

Fig. S3 Thermal variations of refined electron density at the M1-M4 sites (M4 = Sb) in Co_2InSbO_6 and their average from initial fits to the variable temperature powder synchrotron X-ray data. Sb occupancies at the four sites were refined without any overall stoichiometry constraint, and are multiplied by the atomic number of Sb to give approximate electron densities. Dashed lines show ideal values for Co, In, and Sb and their weighted average. Average density points are close to the theoretical value throughout showing that the unconstrained refinements remain close to the real overall electron count for the metals. The following deductions were drawn from the refined electron densities shown in this plot, and were applied as constraints on the final refinements where overall stoichiometry was constrained to the ideal Co_2InSbO_6 composition;

- M4 site has 100% Sb occupancy throughout
- M3 site is occupied 100% by Co while M1 and M2 sites have variable Co/In occupancy at temperatures $300 \leq T \leq 850$ K.
- M2 site is occupied 100% by Co while M1 and M3 sites have variable Co/In occupancy at temperatures $900 \leq T \leq 1073$ K.

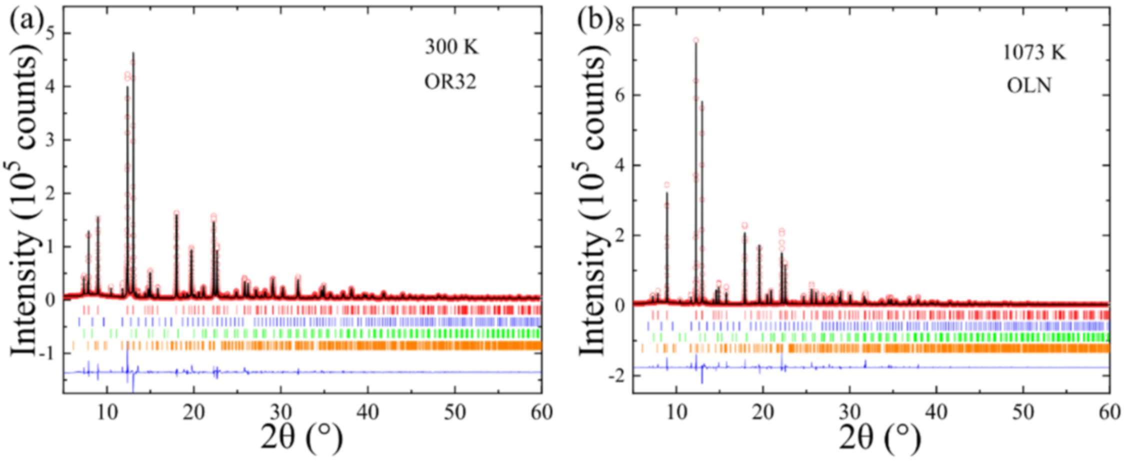

Fig. S4 Final Rietveld refinements of the Co_2InSbO_6 structure (red markers) using SXRD data collected at (a) 300 K and (b) 1073 K. Impurities phases are In_2O_3 (blue markers; (a) 0.9 and (b) 0.7 wt%) and $CoSb_2O_6$ (green markers; (a) 0.8 and (b) 0.6 wt%). Two further small, unidentified peaks were Le Bail fitted as a further phase (orange markers; space group Pbmn, a = 7.94077(1), b = 7.89074(1), c = 4.3829(1) but this indexing is nominal.)

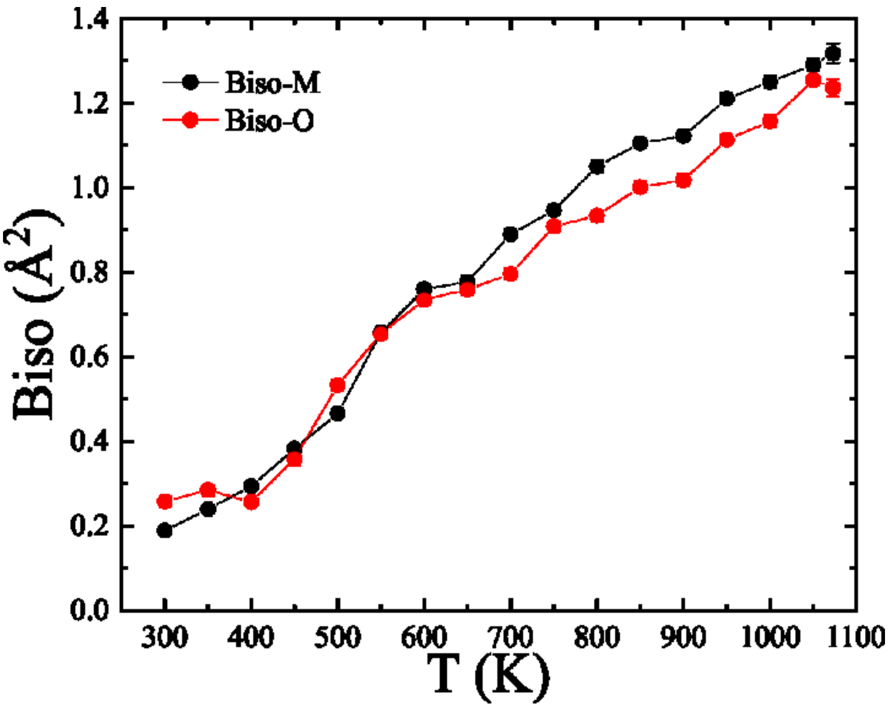

Fig. S5 Thermal variation of the isotropic temperature factors for cations and oxygen atoms from the Co_2InSbO_6 refinements shown in Fig. 3.

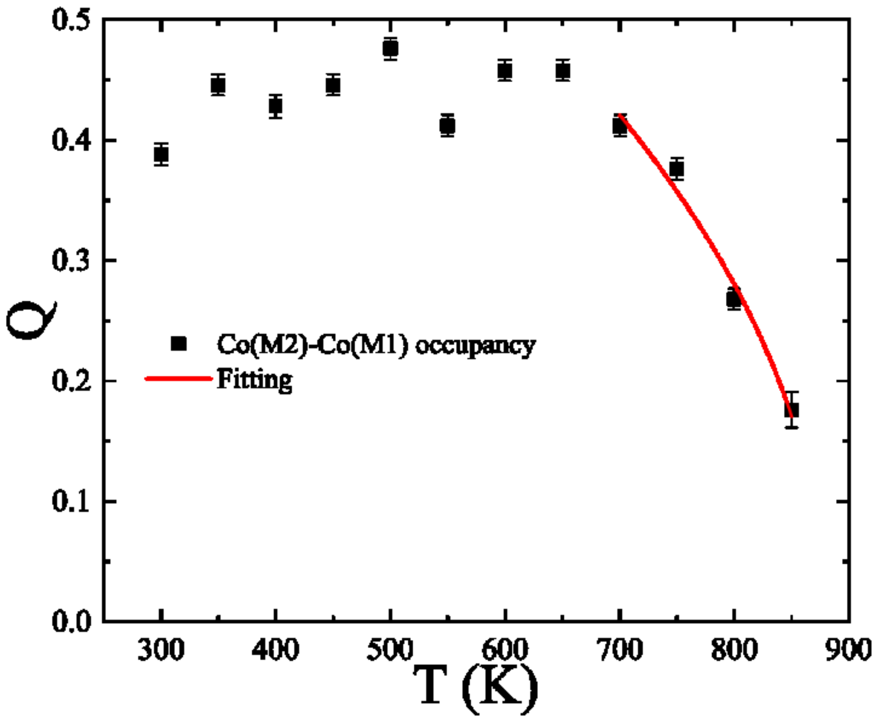

Fig. S6 Plot of Q = Co(M2)-Co(M1) occupancy difference using data up to 850 K from Fig. 3b, with a mean field fit to 700 $\leq T \leq$ 850 K points giving an estimated transition temperature T_{c1} = 879(11) K.

Table S1. Crystallographic results for $(Co_{0.17}In_{0.33}Sb_{0.5})O_2$ from the Rietveld fit of laboratory XRD data collected at 300 K using space group *Pnnm*. Cell parameters are *a* = 4.5502(1) Å, *b* = 5.1024(1) and *c* = 3.1648(1) Å and residuals are R_p=3.32%, R_{wp}=2.38%.

Wyckoff	Atom	x	y	z	Occ	B_{iso}(Å^2)
2a	M1 (Co)	0	0	0	0.17(3)	0.04(1)
2a	M1 (In)	0	0	0	0.33(3)	0.04(1)
2a	M1 (Sb)	0	0	0	0.5	0.04(1)
4g	O	1/3	2/3	0.347(1)	1.0	0.04(1)

Table S2. Crystallographic results for Co_2InSbO_6 (OR32 sample) from the Rietveld fit of SXRD data collected at 300 K using space group *R*3. Sites M3 and M4 were found to be occupied by only one cation in initial refinements and their occupancies were fixed here. Cell parameters are *a* = 5.28813(3) Å and *c* = 14.0294(1) Å and residuals are R_p=6.35%, R_{wp}=9.80%, R_B=5.05%, R_F=2.40%.

Wyckoff	Atom	x	y	z	Occ	B_{iso}(Å^2)
3a	M1 (Co/In)	0.3333	0.6667	0.2792(2)	0.30/0.70(1)	0.19(1)
3a	M2 (Co/In)	0.6667	0.3333	0.4555(6)	0.70/0.30(1)	0.19(1)
3a	M3 (Co)	0	0	0.3221(7)	1.0	0.19(1)
3a	M4 (Sb)	0.3333	0.6667	0.5	1.0	0.19(1)
9b	O1	0.6290(2)	0.6769(4)	0.4055(1)	1.0	0.26(1)
9b	O2	0.0008(2)	0.6371(3)	0.5646(1)	1.0	0.26(1)

Table S3. Crystallographic results for Co_2InSbO_6 from the Rietveld fit of SXRD data collected at 900 K using space group *R*3. Sites M2 and M4 were found to be occupied by only one cation in initial refinements and their occupancies were fixed here. Cell parameters are *a* = 5.30811(1) Å and *c* = 14.13433(7) Å and residuals are R_p=6.86%, R_{wp}=10.3%, R_B=7.60%, R_F=5.15%.

Wyckoff	Atom	x	y	z	Occ	B_{iso}(Å^2)
3a	M1 (Co/In)	0.3333	0.6667	0.2809(1)	0.55/0.45(1)	1.12(1)
3a	M2 (Co)	0.6667	0.3333	0.4527(4)	1.0	1.12(1)
3a	M3 (Co/In)	0	0	0.3236(3)	0.45/0.55(1)	1.12(1)
3a	M4 (Sb)	0.3333	0.6667	0.5	1.0	1.12(1)
9b	O1	0.6231(3)	0.6698(4)	0.4093(1)	1.0	1.02(1)
9b	O2	0.0045(3)	0.6324(3)	0.5611(1)	1.0	1.02(1)

Table S4. Crystallographic results for Co_2InSbO_6 from the Rietveld fit of SXRD data collected at 1073 K using space group *R*3. Sites M2 and M4 were found to be occupied by only one cation in initial refinements and their occupancies were subsequently fixed. Cell parameters are a = 5.31234(2) Å and c = 14.16817(7) Å and residuals are R_p=6.50%, R_{wp}=9.92%, R_B=5.57%, R_F=4.02%.

Wyckoff	Atom	x	y	z	Occ	B_{iso}(Å^2)
3a	M1 (Co/In)	0.3333	0.6667	0.2832(3)	0.83/0.17(1)	1.32(2)
3a	M2 (Co)	0.6667	0.3333	0.4463(3)	1.0	1.32(2)
3a	M3 (Co/In)	0	0	0.3212(3)	0.17/0.83(1)	1.32(2)
3a	M4 (Sb)	0.3333	0.6667	0.5	1.0	1.32(2)
9b	O1	0.6161(3)	0.6643(4)	0.4124(1)	1.0	1.24(2)
9b	O2	0.0093(4)	0.6361(3)	0.5661(1)	1.0	1.24(2)

Table S5. M-O bond lengths calculated from Co_2InSbO_6 structures in Tables S2-S4, and BVS calculations using the 300 K values. The BVS values confirm that sites M2 and M3 are Co-rich while M1 is In-rich following the ordered-R32 A_2BCO_6 structure type in Fig. 1.

	d_{300K}	d_{900K}	d_{1073K}	BVS_{300K}
M1-O	3 x 2.35(2)	3 x 2.37(1)	3 x 2.37(2)	2.72
	3 x 2.03(3)	3 x 2.10(1)	3 x 2.08(2)	
M2-O	3 x 2.28(2)	3 x 2.28(2)	3 x 2.39(1)	2.08
	3 x 2.05(2)	3 x 2.00(1)	3 x 1.97(1)	
M3-O	3 x 2.18(1)	3 x 2.24(1)	3 x 2.31(1)	1.70
	3 x 2.11(2)	3 x 2.17(1)	3 x 2.13(1)	
Sb-O	3 x 2.03(2)	3 x 2.00(2)	3 x 1.97 (2)	5.17
	3 x 1.91(1)	3 x 1.88(1)	3 x 1.90(1)	

[1] J. Rodríguez-Carvajal, *Program included FullProf Suite, version July-2010, ILL* **2010**.
